# ﻿Palynological features and taxonomic significance for 16 species of *Gagea* (Liliaceae) from Xinjiang, China

**DOI:** 10.3897/phytokeys.225.101518

**Published:** 2023-04-21

**Authors:** Musen Lin, Juan Qiu, Kaiqing Xie, Dunyan Tan

**Affiliations:** 1 College of Life Sciences, Xinjiang Agricultural University, Ürümqi 830052, China Xinjiang Agricultural University Ürümqi China; 2 Key Laboratory of Ministry of Education for Western Arid Region Grassland Resources and Ecology, College of Grassland Sciences, Xinjiang Agricultural University, Ürümqi 830052, China Xinjiang Agricultural University Ürümqi China

**Keywords:** HCA, pollen morphology, SEM, taxonomy

## Abstract

Since pollen characters can be used to help distinguish species, our aim was to determine if palynological information has taxonomic significance for *Gagea* species from Xinjiang, China. *Gagea* is widely distributed in north temperate and the subtropical zones. The genus has limited taxonomic characteristics and large morphological variation, which results in difficulty of species classification. Pollen morphology of 16 species of this genus was examined comprehensively via light microscope (LM) and scanning electron microscope (SEM). One qualitative and nine quantitative traits of the pollen grains were surveyed, followed by hierarchical cluster analysis (HCA). The pollen grains were bilaterally symmetrical heteropolar monads with a mono-sulcus and they were oblate or peroblate (Polar diameter (P) / Equatorial diameter (E) = 0.36–0.73) in shape and medium to large (P = 17.17–34.64 μm, E = 27.63–81.65 μm) in size. Three types of exine ornamentation were observed: perforate, microreticulate and reticulate cristatum. The HCA divided the 16 species into two groups. This research provides new data on pollen morphology for *Gagea* (the pollen morphology of eight species was reported for the first time). Pollen morphology also can be used to identify species with similar external morphology, such as *G.nigra* and *G.filiformis*. Furthermore, the study of pollen morphology not only provides new data for palynology research on *Gagea*, but also provides a basis for future classification of this genus.

## ﻿Introduction

Pollen morphology is scarcely affected by ecological conditions; thus, it is more stable than external macrographical morphology and has high genetic stability (Zarre and Zarrei 2005; Blackmore 2007; Kadluczka et al. 2022). Pollen size and morphology, such as shape, aperture type, mesocolpium diameter, exine ornamentation and perforation size, have great significance in plant taxonomy and pollen characteristics can be used to infer genetic relationships between ecological groups (Li et al. 2020) or interspecific taxonomic ranks (Heidarian et al. 2021).

*Gagea* Salisb. (Salisbury 1806) is a genus in tribe Tulipeae of Liliaceae and includes ca. 300 species (Patterson and Givnish 2002; Peruzzi 2016; Peterson et al. 2019). This genus has a wide distribution in regions of South Africa, Asia and Europe (Zarrei et al. 2007; Peterson et al. 2008; Peterson et al. 2019). In Asia, the western Pamir-Alai and western Tien-Shan Mountains are two centres of diversification of *Gagea*, and south-western Asia is the most probable ancestral area for *Gagea*. (Peterson et al. 2009; Peterson et al. 2019; Kurbaniyazova et al. 2022). Species circumscription in *Gagea* is difficult due to overlapping primitive and advanced morphological characters, particularly if only dried specimens are available for study (Zarrei et al. 2009; Tison et al. 2013). To add to the difficulty of identifying species of *Gagea*, there is enormous variation in vegetative and generative characters at various stages of ontogeny under variable ecological conditions (Levichev 1990, 1999) and polyploidy, hybridisation and convergent evolution make species boundaries unclear (Zarrei et al. 2011).

The initial phylogenetic analysis of *Gagea* was conducted in 2008 (Peterson et al. 2008), but the phylogenetic relationship between *Gagea* and *Lloydia* have been controversial. In accordance with the weight given to morphology and/or phylogeny and various molecular markers, various sections of *Gagea* have been suggested (Zarrei et al. 2011). Various molecular studies, based on ITS nrDNA and a distinct plastid dataset (*rbc*L, *ndh*F, *trn*L-*trn*F, *psb*A-*trn*H, *mat*K, *trn*K and *rpl*16 intron) have divided *Gagea* into seven sections (Zarrei et al. 2009), 13 sections (Levichev 2011), 14 sections (Peterson et al. 2008, 2016; Peruzzi 2012) or 15 sections (Levichev 2013; Tison et al. 2013). Molecular data reveal that *Lloydia* is phylogenetically nested within *Gagea*, forming a monophyletic group (Peterson et al. 2008; Zarrei et al. 2009). Therefore, a revision of *Gageasensu lato* (including *Lloydia*) was proposed (Peruzzi et al. 2008; Zarrei et al. 2011) and recent data on pollen characteristics also supported the placement of *Lloydia* within *Gagea* (Hu et al. 2021).

The *Flora of China* includes 17 species of *Gagea* (Chen and Turland 2000); however, three new species from Inner Mongolia, three new species and two new records from Xinjiang recently have been described (Zhao and Zhao 2003, 2004; Zhao and Yang 2006; Peterson et al. 2011). Meanwhile, *G.nigra* was revised, based on morphological characters (Peterson et al. 2011). The Duocet Group (https://duocet.ibiodiversity.net/) moved eight species from *Lloydia* to *Gagea* according to APG IV and recognised that *Gageasensu lato.* Xinjiang is located in northwest China which includes most of the Tien-Shan Mountains. According to statistics, there are 36 species of *Gagea* in China, 21 of which are naturally distributed in Xinjiang. These species are separated by morphological characters of the bulbs, leaves, flowers, fruits and seeds (Chen and Turland 2000; Peterson et al. 2011) and molecular data of cpDNA and nrDNA (Peterson et al. 2008, 2016).

At present, the pollen morphology of 46 species in *Gagea* has been described by various researchers from different regions in the world (Kosenko 1999; Zarre and Zarrei 2005; Wang et al. 2013; Hu et al. 2021; Sezer and Yildiz 2021). These studies demonstrated that shape and exine ornamentation have important information for taxonomic identification amongst species of *Gagea*. Eight species with reported pollen morphology were distributed in Xinjiang, China (Zarre and Zarrei 2005; Wang et al. 2013; Hu et al. 2021), but their taxonomic significance was not thoroughly investigated.

Through extensive field investigations in Xinjiang, we have found that *G.nigra* is similar to *G.filiformis*. The most salient resemblance characters were observed in *G.nigra* and *G.filiformis*, such as ovoid-globose bulb, brown or black tunic, leaf and flower number, umbellate or corymbose inflorescence, yellow tepals, capitate stigma, obovoid capsule and red-brown, ovoid-globose seeds. Furthermore, *G.jensii* shared morphological characters with *G.altaica* and *G.alberti*, including ovoid bulbs, taupe tunic, cauline leaves, corymbose or racemose inflorescence, yellow tepals, slightly 3-lobed stigma and brown flat seeds. Hence, the purpose of this research is to: (1) provide palynological information for 16 species of *Gagea* from Xinjiang, China, using a light microscope (LM) and scanning electron microscope (SEM); (2) distinguish species with similar morphology using palynological characters; and (3) explain the taxonomic significance of palynology in *Gagea*.

## ﻿Materials and methods

### ﻿Pollen materials

During April-July 2020–2022, a field investigation was carried out in Xinjiang, China, including the Altai Mountains, Tien-Shan Mountains, Kunlun Mountains and Junggar Basin to collect samples of pollen. Pollen collections were made from a total of 60 populations of the 16 *Gagea* species in Xinjiang. For widespread species (such as *G.bulbifera*, *G.fedtschenkoana* and *G.nigra*) at least five populations were included in this research. If there were no differences in pollen morphology amongst the populations of a species, one population was selected as a representative for SEM. If populations of a species varied, separate studies were conducted on all of the populations for the species.

The species were identified after collection by comparing their morphological characteristics to those listed in *Flora of China* (Chen and Turland 2000) and Peterson et al. (2011). Standardisation of the scientific names of species was according to Plants of the World Online (https://powo.science.kew.org). The voucher material of 16 species of *Gagea* used for SEM is given in Table 1. All voucher specimens were deposited in the Herbarium of Xinjiang Agricultural University (XJA).

**Table 1.** The list of materials for scanning electron microscope and vouchers of 16 species of *Gagea* from Xinjiang, China.

Species	Section (Peterson et al. 2016)	Locality	Coordinate	Altitude	Collection Date	Voucher
*Gageaalberti* Regel	*Plecostigma*	Shihezi City, Xinjiang, China	44.188091°N, 86.088827°E	517 m	12 April 2022	J.Qiu & J.L.Li L-034 (XJA)
*G.altaica* Schischk. & Sumnev.	*Plecostigma*	Fuyun County, Xinjiang, China	46.368831°N, 88.926181°E	777 m	15 April 2021	J.Qiu & M.S.Lin L-006 (XJA)
*G.angelae* Levichev & Schnittler	*Gagea*	Gongliu County, Xinjiang, China	43.110484°N, 82.751261°E	1660 m	4 May 2021	J.C.Chi Chijc4473 (XJA)
*G.bulbifera* (Pall.) Salisb.	*Bulbiferae*	Shawan County, Xinjiang, China	45.328851°N, 88.455975°E	561 m	7 April 2021	J.Qiu & M.S.Lin L-002 (XJA)
*G.divaricata* Regel	*Platyspermum*	Fukang City, Xinjiang, China	44.739223°N, 88.270266°E	616m	17 April 2021	J.Qiu & M.S.Lin L-011 (XJA)
*G.fedtschenkoana* Pascher	*Gagea*	Qinghe County, Xinjiang, China	46.746946°N, 90.873269°E	2761 m	6 June 2021	J.Qiu & M.S.Lin L-018 (XJA)
*G.filiformis* (Ledeb.) Kar. & Kir.	*Minimae*	Ürümqi City, Xinjiang, China	43.786772°N, 87.565508°E	1075 m	3 April 2021	J.Qiu & M.S.Lin L-004 (XJA)
*G.fragifera* (Vill.) E.Bayer & G.López	*Didymobolbos*	Burqin City, Xinjiang, China	48.429559°N, 87.207309°E	1988 m	9 June 2021	J.Qiu & M.S.Lin L-021 (XJA)
*G.granulosa* Turcz.	*Minimae*	Burqin City, Xinjiang, China	48.429694°N, 87.207108°E	1984 m	9 June 2021	J.Qiu & M.S.Lin L-023 (XJA)
*G.jaeschkei* Pascher	*Bulbiferae*	Qapqal County, Xinjiang, China	43.411647°N, 81.040648°E	2929 m	17 July 2021	J.Qiu & M.S.Lin L-30 (XJA)
*G.jensii* Levichev & Schnittler	*Plecostigma*	Ürümqi City, Xinjiang, China	43.783443°N, 87.544818°E	1002 m	8 April 2021	J.Qiu & M.S.Lin L-005 (XJA)
*G.nigra* L.Z.Shue	*Minimae*	Ürümqi City, Xinjiang, China	43.783141°N, 87.544363°E	995 m	2 April 2021	J.Qiu & M.S.Lin L-003 (XJA)
*G.neopopovii* Golosk.	*Plecostigma*	Huocheng County, Xinjiang, China	44.483283°N, 81.175174°E	2100 m	19 May 2021	X.J. Ge Gexj-21019 (XJA)
*G.kunawurensis* (Royle) Greuter	*Dschungaricae*	Ürümqi City, Xinjiang, China	43.785813°N, 87.545323°E	997 m	20 April 2021	J.Qiu & M.S.Lin L-015 (XJA)
*G.stepposa* L.Z.Shue	*Bulbiferae*	Ürümqi County, Xinjiang, China	43.516102°N, 87.447984°E	1559 m	10 April 2022	J.Qiu & J.L.Li L-032 (XJA)
*G.tenera* Pascher	*Didymobolbos*	Nilka County, Xinjiang, China	43.724538°N, 82.070252°E	1033 m	22 April 2022	J.Qiu & J.L.Li L-041 (XJA)

### ﻿Preparation and observation for pollen slides

All pollen grains were taken from fresh flowers, except for *G.angelae* and *G.neopopovii*, which were obtained from herbarium specimens. At peak flowering in each natural population, five individual plants were selected for pollen collection. Mature anthers were removed before dehiscence and placed in a clean glass bottle for natural drying.

Pollen slides were prepared following standard methods (Erdtman 1960) and observed under LM and investigated with the SEM following the methods of Halbritter et al. (2018). Mature pollen grains were mounted directly on dusted stubs with double-coated conductive glue and coated with gold-palladium, then examined under a Zeiss SUPRA 55VP (Carl Zeiss, Oberkochen, Germany). Terminology for the description of pollen follows Erdtman (1969), Punt et al. (2007) and Halbritter et al. (2018).

A label that referred to the number of the voucher specimen was attached to each slide. Pollen grains were photographed under LM (Nikon Eclipse 80i) at a magnification of 40× and SEM (Zeiss SUPRA 55VP) at an accelerating voltage of 2 kV. Each species/ population observed the polar diameter (P) and equatorial diameter (E) of 30 pollen grains under LM with an immersion objective lens (at 100× magnification). The microscopic morphological features of pollen [colpus width (Clt), colpus length (Clg), porus width (Plt), porus length (Plg), exine thickness (Ex), intine thickness (In) and ornamentation] were measured under SEM.

### ﻿Data analysis

The results of palynological measurements were evaluated by statistical analysis and the contribution of each variable to the classification of each investigated was determined.

A one-way ANOVA was used to determine differences in pollen morphology of different populations of the same species. Prior to the analysis, the SPSS programme version 26 was used to test for normality and homogeneity of variance to satisfy the requirements of one-way analysis of variance (ANOVA). Differences amongst species were determined by the non-parametric Kruskal-Wallis test.

One qualitative (ornamentation) and nine quantitative palynological variables (polar diameter, equatorial diameter, P/ E, porus length, porus width, colpus length, colpus width, exine thickness and intine thickness) were evaluated in the comparative analysis for their value in distinguishing the studied *Gagea* species. Quantitative variables were represented by minimum (mean ± standard error) and maximum (for example: 20.34 (20.64 ± 0.24) 21.12 μm), whereas qualitative variables were recorded in the data matrix as 0, 1 or 2. Origin 2021 software was adopted for hierarchical cluster analysis (HCA) on *Gagea* pollen data. Euclidean distances of the stem were calculated after Z-score normalizing the original data and they were clustered using Ward’s method (Ye et al. 2015).

## ﻿Results

Within populations of each species, pollen shape and exine ornamentation were stable, but the size of pollen grains was not. One-way ANOVA showed that the means of equatorial diameter were not significantly different amongst populations of the same species (*p* > 0.05). Nevertheless, there was a significant difference in mean equatorial diameter amongst different species (H = 378.016, df = 15, *p* < 0.001). Therefore, a random population was selected as a representative material for each species for SEM and the micro-morphological characters of pollen grains were carefully observed.

**Figure 1.**
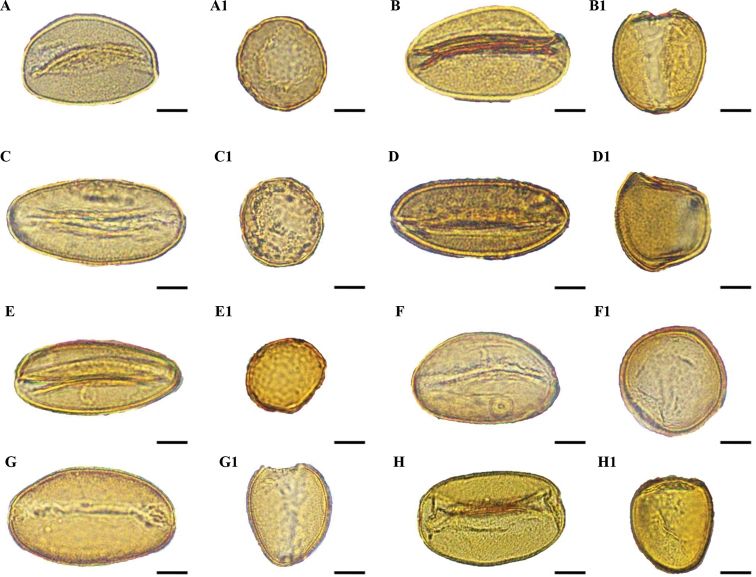
Light microscope micrographs of pollen grains of eight species of *Gagea* from Xinjiang, China **A, A1***Gageaalberti***B, B1***G.altaica***C, C1***G.angelae***D, D1***G.bulbifera***E, E1***G.divaricata***F, F1***G.fedtschenkoana***G, G1***G.filiformis***H, H1***G.fragifera*. **A–H** Pollen grain in polar view **A1–H1** Pollen grain in equatorial view. Scale bars: 10 μm.

**Figure 2.**
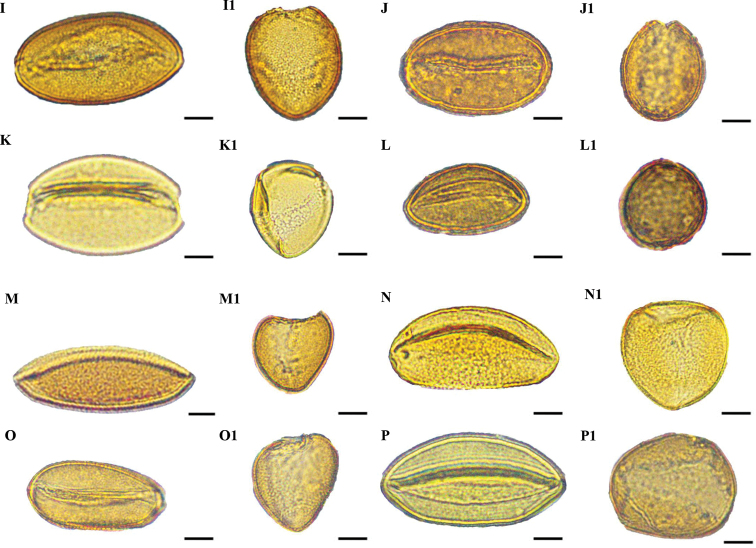
Light microscope micrographs of pollen grains of eight species of *Gagea* from Xinjiang, China. **I, I1***Gageagranulosa***J, J1***G.jaeschkei***K, K1***G.jensii***L, L1***G.neopopovii***M, M1***G.nigra***N, N1***G.kunawurensis***O, O1***G.stepposa***P, P1***G.tenera*. **I–P** Pollen grain in polar view. **I1–P1** Pollen grain in equatorial view. Scale bars: 10 μm.

**Figure 3.**
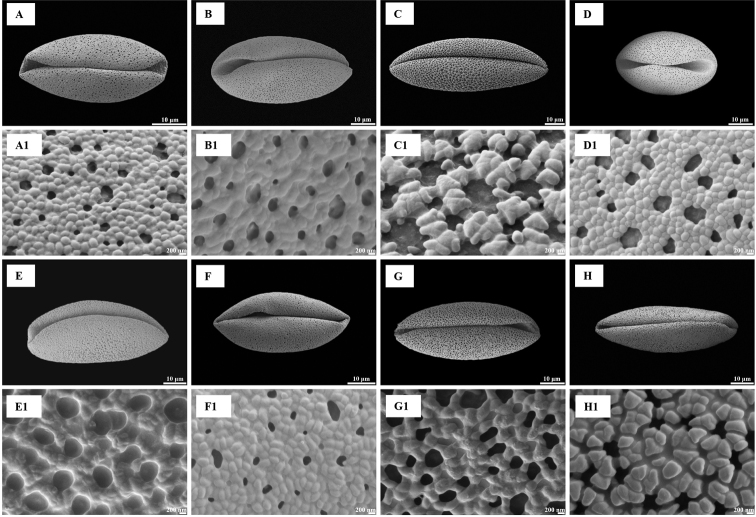
Pollen grains in polar view (**A–H**) and exine ornamentation (**A1–H1**) under scanning electron microscope for eight species of *Gagea* from Xinjiang, China **A, A1***Gageaalberti***B, B1***G.altaica***C, C1***G.angelae***D, D1***G.bulbifera***E, E1***G.divaricata***F, F1***G.fedtschenkoana***G, G1***G.filiformis***H, H1***G.fragifera*.

**Figure 4.**
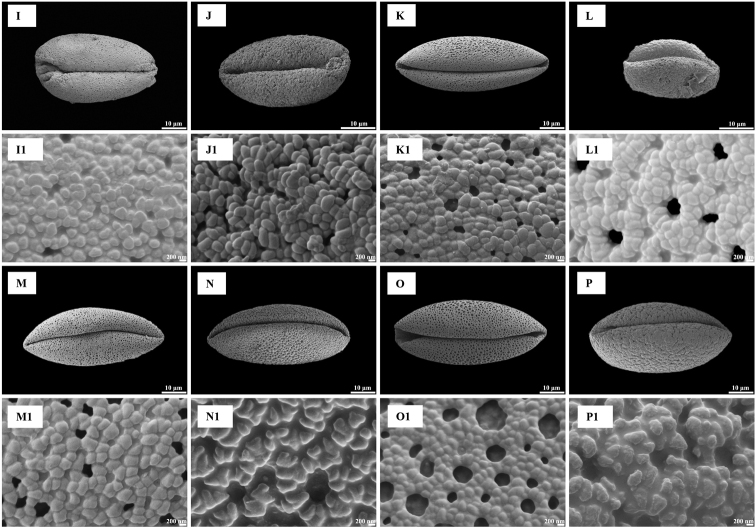
Pollen grains in polar view (**I–P**) and exine ornamentation (**I1–P1**) under scanning electron microscope for eight species of *Gagea* from Xinjiang, China **I, I1***Gageagranulosa***J, J1***G.jaeschkei***K, K1***G.jensii***L, L1***G.neopopovii***M, M1***G.nigra***N, N1***G.kunawurensis***O, O1***G.stepposa***P, P1***G.tenera*.

In general, pollen grains of the *Gagea* species were similar in their morphological characters. The detailed pollen morphology data for the 16 species of *Gagea* are summarised in Table 2. Representative pollen grains of LM and SEM micrographs are shown in Figs 1–4.

**Table 2.** Pollen morphology of 16 species of *Gagea* from Xinjiang, China.

Species	P (μm) Min (Mean ± SE) Max	E (μm) Min (Mean ± SE) Max	P/E	Plg (μm) Min (Mean ± SE) Max	Plt (μm) Min (Mean ± SE) Max
*Gageaalberti*	20.34 (20.64±0.24) 21.12	42.77 (45.73±1.85) 49.14	0.45	0.28 (0.39±0.05) 0.72	0.24 (0.37±0.03) 0.57
*G.altaica*	27.93 (29.34±0.55) 30.61	57.32 (62.41±3.69) 73.36	0.47	0.24 (0.39±0.03) 0.58	0.21 (0.37±0.03) 0.49
*G.angelae*	26.38 (28.18±1.07) 30.07	71.97 (78.14±3.09) 81.65	0.36	0.46 (0.75±0.06) 1.04	0.42 (0.57±0.03) 0.71
*G.bulbifera*	26.28 (30.92±0.92) 34.64	36.73 (42.71±1.34) 51.34	0.73	0.16 (0.50±0.02) 0.49	0.14 (0.37±0.03) 0.43
*G.divaricata*	28.11 (28.56±0.40) 29.36	46.30 (56.86±5.30) 62.91	0.49	0.58 (0.72±0.08) 0.94	0.54 (0.68±0.11) 1.10
*G.fedtschenkoana*	22.37 (22.74±0.19) 23.02	49.12 (58.22±5.52) 68.18	0.41	0.13 (0.36±0.09) 0.62	0.13 (0.23±0.03) 0.33
*G.filiformis*	23.72 (24.58±0.41) 25.75	58.94 (62.60±2.21) 71.08	0.39	0.36 (0.45±0.05) 0.56	0.28 (0.40±0.06) 0.54
*G.fragifera*	20.98 (23.78±1.41) 25.50	62.22 (65.61±2.01) 69.16	0.36	0.44 (0.53±0.03) 0.63	0.29 (0.39±0.04) 0.50
*G.granulosa*	19.58 (25.50±2.96) 28.63	49.84 (51.89±1.04) 53.23	0.49	0.19 (0.30±0.02) 0.36	0.15 (0.22±0.02) 0.32
*G.jaeschkei*	15.07 (20.10±2.65) 29.43	33.36 (37.53±1.11) 39.43	0.54	0.12 (0.20±0.02) 0.26	0.06 (0.14±0.02) 0.26
*G.jensii*	22.86 (23.28±0.82) 25.80	46.89 (53.34±2.63) 62.16	0.44	0.13 (0.22±0.03) 0.44	0.10 (0.18±0.02) 0.33
*G.neopopovii*	17.17 (22.15±0.99) 26.13	27.63 (44.79±4.32) 66.88	0.54	0.30 (0.44±0.02) 0.69	0.15 (0.31±0.02) 0.62
*G.nigra*	20.26 (22.50±1.16) 24.14	41.77 (51.41±4.86) 57.35	0.45	0.27 (0.38±0.06) 0.55	0.13 (0.34±0.05) 0.42
*G.kunawurensis*	21.92 (23.58±0.88) 24.94	50.66 (56.82±3.20) 61.42	0.41	0.58 (0.78±0.05) 1.00	0.43 (0.64±0.03) 0.76
*G.stepposa*	25.31 (26.15±0.43) 26.68	58.51 (61.87±1.90) 65.10	0.42	0.28 (0.46±0.05) 0.73	0.17 (0.39±0.05) 0.63
*G.tenera*	18.34 (23.14±2.43) 29.17	42.89 (48.63±3.47) 58.31	0.47	0.28 (0.43±0.04) 0.63	0.18 (0.33±0.04) 0.52
**Species**	**Clg (μm) Min (Mean** ± **SE) Max**	**Clt (μm) Min (Mean** ± **SE) Max**	**Ex (μm) Min (Mean** ± **SE) Max**	**In (μm) Min (Mean** ± **SE) Max**	**Ornamentation**
*Gageaalberti*	41.35 (44.38±1.95) 48.02	1.67 (1.95±0.19) 2.32	0.93 (1.57±0.18) 2.93	0.50 (0.90±0.09) 1.45	Perforate (0)
*G.altaica*	56.01 (60.35±3.36) 70.38	3.25 (3.61±0.38) 4.01	0.81 (1.59±0.07) 2.08	0.58 (0.97±0.04) 1.39	Microreticulate (1)
*G.angelae*	69.65 (75.38±2.87) 78.32	0.87 (1.60±0.47) 2.48	0.88 (1.83±0.12) 2.42	0.61 (0.99±0.05) 1.29	Reticulate cristatum (2)
*G.bulbifera*	36.47 (42.05±2.02) 48.24	1.71 (2.14±0.23) 2.78	1.41 (1.90±0.08) 2.63	0.78 (1.16±0.07) 1.93	Microreticulate (1)
*G.divaricata*	43.60 (53.08±4.74) 57.84	3.74 (3.95±0.19) 4.33	1.11 (1.65±0.08) 2.26	0.51 (0.81±0.06) 1.42	Reticulate cristatum (2)
*G.fedtschenkoana*	47.10 (56.07±5.62) 66.42	1.51 (1.60±0.06) 1.71	1.32 (1.77±0.07) 2.17	0.72 (0.92±0.03) 1.14	Microreticulate (1)
*G.filiformis*	55.51 (59.02±2.45) 68.68	1.96 (2.17±0.13) 2.71	0.47 (1.41±0.12) 2.35	0.28 (0.75±0.06) 1.14	Microreticulate (1)
*G.fragifera*	57.64 (61.54±2.80) 66.98	1.85 (2.00±0.08) 2.12	0.76 (1.66±0.11) 2.16	0.39 (0.87±0.06) 1.38	Reticulate cristatum (2)
*G.granulosa*	46.19 (49.36±1.62) 51.54	2.04 (2.70±0.56) 3.81	1.72 (2.30±0.10) 2.73	0.94 (1.23±0.06) 1.57	Perforate (0)
*G.jaeschkei*	30.47 (34.31±1.01) 36.46	0.73 (1.54±0.27) 2.36	1.02 (1.94±0.13) 2.36	0.56 (1.04±0.08) 1.40	Perforate (0)
*G.jensii*	44.45 (51.18±2.70) 60.38	1.64 (2.59±0.29) 3.09	1.22 (1.49±0.18) 2.18	0.76 (1.06±0.14) 1.57	Perforate (0)
*G.neopopovii*	24.98 (41.03±4.53) 63.26	1.13 (1.30±0.06) 1.83	1.00 (1.79±0.20) 2.70	0.64 (1.04±0.14) 1.57	Reticulate cristatum (2)
*G.nigra*	39.05 (48.80±4.97) 55.39	0.92 (1.24±0.30) 1.85	1.12 (1.59±0.09) 2.24	0.53 (0.83±0.05) 1.12	Reticulate cristatum (2)
*G.kunawurensis*	48.20 (54.48±3.34) 59.61	1.18 (1.62±0.33) 2.26	1.34 (1.97±0.08) 2.51	0.78 (1.14±0.05) 1.46	Reticulate cristatum (2)
*G.stepposa*	57.46 (60.59±1.67) 63.19	2.30 (2.55±0.14) 2.78	0.66 (1.31±0.10) 1.85	0.27 (0.72±0.07) 1.32	Microreticulate (1)
*G.tenera*	38.48 (44.19±3.38) 53.58	0.54 (1.01±0.30) 1.87	1.08 (2.10±0.13) 2.89	0.44 (1.07±0.08) 1.48	Reticulate cristatum (2)

Abbreviations: P: Polar diameter; E: Equatorial diameter; Plg: Porus length; Plt: Porus width; Clg: Colpus length; Clt: Colpus width; Ex: Exine thickness; In: Intine thickness.

### ﻿Shape and size

Pollen grains of all species were heteropolar monads and they were mono-sulcus, bilaterally symmetrical and ellipsoidal in polar view. Pollen shape was oblate (Figs 1D, 2J, L) or peroblate (Figs 1A–C, E–H, 2I, K, M, N–P), based on the P/E ranges (Table 2). Pollen size was medium (Figs 3A, D, 4J, L, P) to large (Figs 3B, C, E, F–H, 4I, K, M–O), based on measurements of the equatorial diameter (Table 2).

### ﻿Exine, intine and ornamentation

Thickness of the pollen exine and intine of all species was similar, ranging from 1.31 ± 0.10 to 2.3 ± 0.1 μm and from 0.72 ± 0.07 to 1.23 ± 0.06 μm, respectively. Only *G.stepposa* had a thinner exine than the other species (Table 2).

Three types of pollen grain exine ornamentation were identified: type I, Perforate (Figs 3A1, 4I1, J1, K1), type II, Microreticulate (Figs 3B1, D1, F1, G1, 4O1) and type III, Reticulate cristatum (Figs 3C1, E1, H1, 4L1, M1, N1, P1).

In type I, exine ornamentation had gemmate protuberances. Perforations were smaller than muri and observed throughout the pollen grains. This type was found in *G.alberti*, *G.granulosa*, *G.jaeschkei* and *G.jensii*. The smallest pollen grains were in *G.jaeschkei* with a size (i.e. polar diameter × equatorial diameter) of 20.10 ± 2.65 × 37.53 ± 1.11 μm and the largest pollen grains in *G.jensii* with a size of 23.28 ± 0.82 × 53.34 ± 2.63 μm.

In type II, lumina were not similar in diameter and they were as wide as the muri or smaller than the muri. Muri were complete or compound and the width was narrow from the proximal to distal surface. This type was found in *G.altaica*, *G.bulbifera*, *G.fedtschenkoana*, *G.filiformis* and *G.stepposa*. The smallest pollen grains were discovered in *G.bulbifera* with a size of 30.92 ± 0.92 × 42.71 ± 1.34 μm and the largest pollen grains in *G.filiformis* with a size of 24.58 ± 0.41 × 62.6 ± 2.21 μm.

In type III, lumina were similar in diameter, they were as wide as the muri or wider than the muri. Muri had regular prominent croton pattern or gemmate suprasculpture. Type III was found in *G.angelae*, *G.divaricata*, *G.fragifera*, *G.neopopovii*, *G.nigra*, *G.kunawurensis* and *G.tenera*. The smallest pollen grains were for *G.neopopovii* with a size of 22.15 ± 0.99 × 44.79 ± 4.32 μm and the largest pollen grains in *G.angelae* with a size of 28.18 ± 1.07 × 78.14 ± 3.09 μm.

### ﻿Hierarchical cluster analysis (HCA)

**Figure 5.**
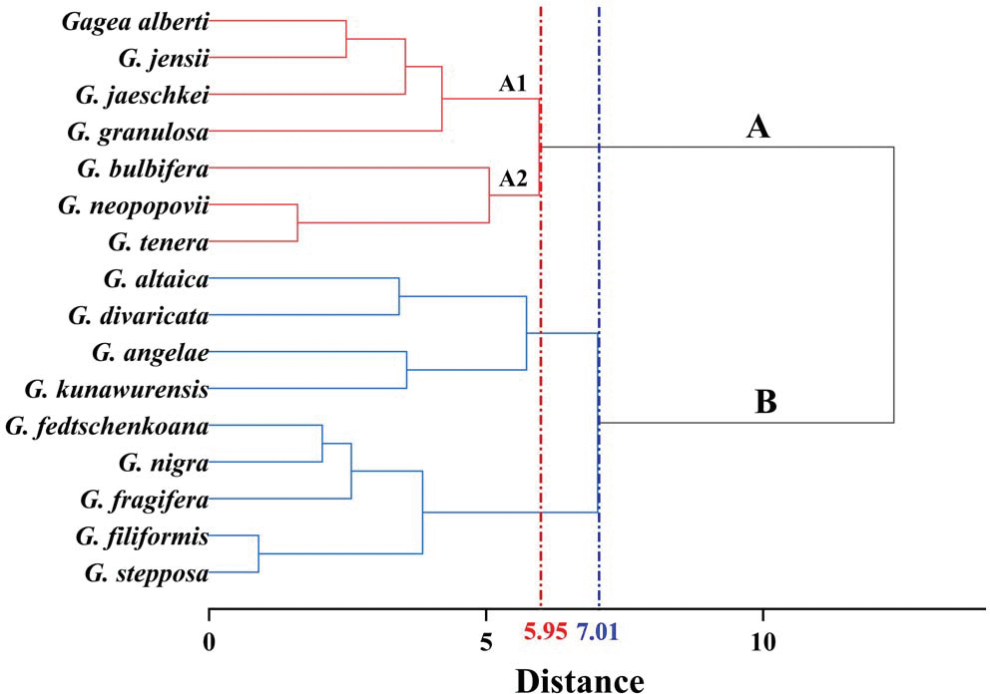
Cluster diagram (Ward’s linkage) of 16 species in *Gagea* from Xinjiang, China, based on one qualitative and nine quantitative pollen characters.

The palynological groups of the species, based on their relevance, were evaluated by hierarchical cluster analysis. In this analysis, pollen morphology separated the *Gagea* species into two groups, based on Euclidean distance of 7.01. Group A included *G.alberti*, *G.bulbifera*, *G.granulosa*, *G.jaeschkei*, *G.jensii*, *G.neopopovii* and *G.tenera*. It was arranged into two subgroups (A1 and A2), based on Euclidean distance of 5.95. Whereas group B involved *G.altaica*, *G.angelae*, *G.divaricata*, *G.fedtschenkoana*, *G.filiformis*, *G.fragifera*, *G.kunawurensis*, *G.nigra* and *G.stepposa* (Fig. 5).

## ﻿Discussion

Our results supported previous research showing that the pollen grains in *Gagea* were bilaterally symmetrical heteropolar monads with a mono-sulcus (Kosenko 1999; Su et al. 2004; Zarre and Zarrei 2005; Wang et al. 2013; Hu et al. 2021; Sezer and Yildiz 2021). Grain exine ornamentation was divergent amongst the species and often served as a diagnostic character (Chung et al. 2010). Additionally, exine ornamentation was one of the most variable characters in *Gagea* pollen, with three types observed in our research (Table 2). Zarre and Zarrei (2005) recognised four pollen exine ornamentation types within *Gagea* and *G.bulbifera* and *G.tenera* were described as microreticulate and reticulate, respectively and matched type II (microreticulate) and type III (reticulate cristatum) of our research, respectively (Table 2). Pollen exine ornamentation of *G.ova* (synonym of *G.kunawurensis*) was described as foveolate (Zarre and Zarrei 2005), differing from type III (reticulate cristatum) found in our research. This result remains to be further confirmed due to lack of photographs in literature.

Pollen morphology of five *Gagea* species (*G.alberti*, *G.bulbifera*, *G.fedtschenkoana*, *G.granulosa* and *G.nigra*) collected from Xinjiang have been described (Wang et al. 2013; Hu et al. 2021). Wang et al. (2013) and Hu et al. (2021) described the exine ornamentation of *G.fedtschenkoana* pollen as verrucate, whereas we described it as perforate (Table 2). Moreover, the exine ornamentation of *G.filiformis* and *G.granulosa* collected from other regions was described as rugulate-perforate and reticulate, respectively (Hu et al. 2021), whereas the exine ornamentation of *G.filiformis* and *G.granulosa* described in our research was microreticulate and perforate, respectively (Table 2). Our results are consistent with those in literature when we compare our results with the published figures and the divergence in descriptions might be due to variations in terminology. Overall, the available data provide compelling evidence that pollen morphology exhibits high genetic stability.

Pollen characters have been employed as useful morphological features for the identification of species or genera and they have been applied widely to *Allium* (Nour et al. 2022), *Praxelis* (de Abreu et al. 2015), *Fritillaria* (Samaropoulou et al. 2022) and *Lythrum* (Vieira et al. 2022) to elucidate intricate taxonomic relationships. Moreover, pollen characters are effective and valuable in distinguishing species with similar morphological features (Ahmad et al. 2022). The pollen of *G.nigra* and *G.filiformis*; *G.jensii*, *G.altaica* and *G.alberti* displayed differences in morphological characters that could be used as identification tools, while the applicability of other morphological characters, such as shape of bulb, leaves, inflorescence, stigma and seeds, colour of tunic, tepals and seeds, number of leaves and flowers, for species identification is restricted. Pollen grains of the above five species were large and peroblate and only the pollen grains of *G.alberti* were medium in size (Table 2). In addition, the aperture type, porus size and pollen wall thickness of these species were similar between species. *Gageanigra* pollen had reticulate cristatum exine ornamentation, whereas that of *G.filiformis* was microreticulate (Figs 3G1, 4M1). *G.jensii* and *G.alberti* pollen had perforate exine ornamentation, whereas that of *G.altaica* was microreticulate (Figs 3A1, B1, 4K1). Despite *G.jensii* and *G.alberti* pollen grains having similar exine ornamentation, they differed in size. (Table 2). Pollen size should be evaluated with circumspection, because it is known to be influenced by biological factors (Rahmawati et al. 2019).

Information on palynology should be used to provide new insight into the differences (or not) between species. For example, in the revision of *G.nigra* by Peterson et al. (2011) that also includes a description of three new species (including *G.jensii* and a comparison of the morphology of *G.jensii* and *G.alberti*), the morphology of *G.jensii* differs from that of *G.alberti*. However, we found that the pollen morphology of *G.jensii* and *G.alberti* is the same (Figs 3A1, 4K1), suggesting that they are possibly the same species. Thus, we suggest that verification of *G.jensii* and *G.alberti* as separate species must be further investigated using cytological, molecular phylogenetic or anatomical techniques. Our research provides a new way to diagnosis species with similar morphological characteristics, but they are not exhaustive.

According to recent infrageneric classification of *Gagea*, the species in this research belong to seven sections (Table 1) (Peterson et al. 2016). HCA was performed on the palynological data obtained from both LM and SEM. Group A included two subgroups (A1, A2) and contained three species of sect. *Plecostigma* (*G.alberti*, *G.jensii* and *G.neopopovii*), two species of sect. *Bulbiferae* (*G.bulbifera* and *G.jaeschkei*) and one species of sect. *Minimae* (*G.granulosa*), sect. *Didymobolbos* (*G.tenera*). The subgroup A1 enclosed four species with perforate exine ornamentation, while the subgroup A2 involved three species with medium size (Fig. 5, Table 2). Group B, which represents the large-sized and oblate-shaped pollen grains, encompassed the following sections: sect. *Plecostigma* (*G.altaica*), sect. *Bulbiferae* (*G.stepposa*), sect. *Didymobulbos* (*G.fragifera*), sect. *Dschungaricae* (*G.kunawurensis*), sect. *Gagea* (*G.angelae* and *G.fedtschenkoana*), sect. *Minimae* (*G.filiformis* and *G.nigra*) and sect. *Platyspermum* (*G.divaricata*) (Fig. 5, Table 2). Thus, the results of the HCA are equivocal as the subgroups contain species from both the same and different sections.

*Gageanigra* and *G.filiformis* belong to the sect. *Minimae*, and morphology and molecular evidence suggest that *G.nigra* is an independent species separated from *G.filiformis* (Peterson et al. 2011). We have also confirmed this view through palynology results, based on two species clustered into one group (Fig. 5). In contrast, *G.altaica*, *G.jensii* and *G.alberti* belong to the sect. *Plecostigma*. However, *G.altaica* separated into a different group with *G.jensii* and *G.alberti*, based on palynology (Fig. 5). This may indicate that there are pollen synapomorphies related to the systematics in certain sections, but not necessarily in all sections.

Although the results are not able to offer a diagnostic key amongst groups of *Gagea* taxa in Xinjiang, China, they demonstrate that palynology may aid in the taxonomy of the genus by differentiating between taxa within their groups.

## ﻿Conclusion

Pollen morphology of eight species of *Gagea* from Xinjiang, China was reported for the first time in our research. *Gagea* pollen grains were heterogenous in shape, size and exine ornamentation. Pollen characters have a certain taxonomic effect on the interspecies of *Gagea*, but the taxonomic relationship cannot be fully clarified only by pollen morphology. The results of the current research have provided palynological data for the classification of *Gagea* and also contribute to future classification of this genus.

## Supplementary materials

Supplementary material 1 Species collection table and a one-way ANOVA results of species with more than two populations Data type pdf file https://binary.pensoft.net/file/840807This dataset is made available under the Open Database License (http://opendatacommons.org/licenses/odbl/1.0/). The Open Database License (ODbL) is a license agreement intended to allow users to freely share, modify, and use this Dataset while maintaining this same freedom for others, provided that the original source and author(s) are credited.Musen Lin, Juan Qiu, Kaiqing Xie, Dunyan Tan
