# Supplementary material for: ﻿Palynological features and taxonomic significance for 16 species of Gagea (Liliaceae) from Xinjiang, China
Source: PhytoKeys. 2023 Apr 21;225:53–68. doi: 10.3897/phytokeys.225.101518 (PMC10194809; doi:10.3897/phytokeys.225.101518)
Supplement: Supplementary material 1 — Species collection table and a one-way ANOVA results of species with more than two populations [file phytokeys-225-053_article-101518__-s001.pdf]

Appendix 1. The list of materials examined and vouchers of 60 populations belonging to 16 species of *Gagea* from Xinjiang, China.

Species	Locality	Coordinate	Altitude	Collection Date	Voucher
Gagea alberti (1)	Urumqi City, Xinjiang, China	45.844092°N, 84.775051°E	927 m	29 April 2020	J.Qiu T-003 (XJA)
G. alberti (2)	Shihezi City, Xinjiang, China	44.188091°N, 86.088827°E	517 m	12 April 2022	J.Qiu & J.L.Li L-034 (XJA)
G. alberti (3)	Urumqi City, Xinjiang, China	43.857741°N, 87.544662°E	795 m	16 April 2022	J.Qiu & J.L.Li L-039 (XJA)
G. alberti (4)	Huocheng County, Xinjiang, China	44.004373°N, 80.936525°E	599 m	21 April 2022	J.Qiu & J.L.Li L-054 (XJA)
G. alberti (5)	Yining City, Xinjiang, China	43.724521°N, 82.070256°E	1033 m	22 April 2022	J.Qiu & J.L.Li L-062 (XJA)
G. altaica (1)	Fuyun County, Xinjiang, China	46.368831°N, 88.926181°E	777 m	15 April 2021	J.Qiu & M.S.Lin L-006 (XJA)
G. altaica (2)	Fuyun County, Xinjiang, China	46.645528°N, 88.593935°E	793 m	16 April 2021	J.Qiu & M.S.Lin L-007 (XJA)
G. altaica (3)	Fuyun County, Xinjiang, China	46.830949°N, 88.791038°E	703 m	16 April 2021	J.Qiu & M.S.Lin L-009 (XJA)
G. angelae	Gongliu County, Xinjiang, China	43.110484°N, 82.751261°E	1660 m	4 May 2021	J.C.Chi Chijc4473 (XJA)
G. bulbifera (1)	Shawan City, Xinjiang, China	45.328851°N, 88.455975°E	561 m	7 April 2021	J.Qiu & M.S.Lin L-002 (XJA)
G. bulbifera (2)	Fuyun County, Xinjiang, China	46.841351°N, 89.405816°E	1028 m	16 April 2021	J.Qiu & M.S.Lin L-008 (XJA)
G. bulbifera (3)	Urumqi City, Xinjiang, China	43.830091°N, 87.780818°E	996 m	18 April 2021	J.Qiu & M.S.Lin L-013 (XJA)
G. bulbifera (4)	Shihezi City, Xinjiang, China	44.194476°N, 86.079483°E	664 m	12 April 2022	J.Qiu & J.L.Li L-033 (XJA)
G. bulbifera (5)	Huocheng County, Xinjiang, China	44.004373°N, 80.936525°E	599 m	21 April 2022	J.Qiu & J.L.Li L-055 (XJA)
G. bulbifera (6)	Yining City, Xinjiang, China	43.817749°N, 81.902141°E	880 m	22 April 2022	J.Qiu & J.L.Li L-058 (XJA)
G. bulbifera (7)	Shawan City, Xinjiang, China	44.235896°N, 85.821398°E	903 m	20 April 2022	J.Qiu & J.L.Li L-053 (XJA)

G. divaricata (1)	Fuhai County, Xinjiang, China	45.053634°N, 88.398225°E	712 m	17 April 2021	J.Qiu & M.S.Lin L-010 (XJA)
G. divaricata (2)	Fukang City, Xinjiang, China	44.739223°N, 88.270266°E	616 m	17 April 2021	J.Qiu & M.S.Lin L-011 (XJA)
G. divaricata (3)	Huocheng County, Xinjiang, China	44.018193°N, 80.775512°E	607 m	21 April 2022	J.Qiu & J.L.Li L-056 (XJA)
G. fedtschenkoana (1)	Nilka County, Xinjiang, China	43.652369°N, 84.362144°E	2278 m	2 June 2020	J.Qiu NLK-003 (XJA)
G. fedtschenkoana (2)	Bole City, Xinjiang, China	44.632994°N, 81.329544°E	2091 m	3 June 2020	J.Qiu SH-001 (XJA)
G. fedtschenkoana (3)	Qinghe County, Xinjiang, China	46.746946°N, 90.873269°E	2761 m	6 June 2021	J.Qiu & M.S.Lin L-018 (XJA)
G. fedtschenkoana (4)	Burqin County, Xinjiang, China	48.504169°N, 87.138269°E	1441 m	9 June 2021	J.Qiu & M.S.Lin L-022 (XJA)
G. fedtschenkoana (5)	Hutubi County County, Xinjiang, China	43.880653°N, 86.480528°E	1151 m	20 April 2022	J.Qiu & J.L.Li L-045 (XJA)
G. fedtschenkoana (6)	Manas County, Xinjiang, China	43.865031°N, 86.252183°E	1526 m	20 April 2022	J.Qiu & J.L.Li
G. filiformis (1)	Burqin County, Xinjiang, China	48.286741°N, 87.093257°E	1352 m	15 April 2020	J.Qiu BEJ-004 (XJA)
G. filiformis (2)	Urumqi City, Xinjiang, China	43.786772°N, 87.565508°E	1075 m	3 April 2021	J.Qiu & M.S.Lin L-004 (XJA)
G. filiformis (3)	Yuming County, Xinjiang, China	45.842106°N, 82.525295°E	1676 m	28 April 2021	J.Qiu Yu-002 (XJA)
G. filiformis (4)	Burqin County, Xinjiang, China	48.430501°N, 87.210654°E	1987 m	9 June 2021	J.Qiu & M.S.Lin L-025 (XJA)
G. filiformis (5)	Manas County, Xinjiang, China	43.865031°N, 86.252183°E	1526 m	20 April 2022	J.L.Li L-049 (XJA)
G. fragifera (1)	Qinghe County, Xinjiang, China	46.781335°N, 90.887534°E	2662 m	6 June 2021	J.Qiu & M.S.Lin L-017 (XJA)
G. fragifera (2)	Fuhai County, Xinjiang, China	48.059008°N, 88.920102°E	2401 m	7 April 2021	J.Qiu & M.S.Lin L-019 (XJA)
G. fragifera (3)	Burqin County, Xinjiang, China	48.429559°N, 87.207309°E	1988 m	9 June 2021	J.Qiu & M.S.Lin L-021 (XJA)

G. granulosa (1)	Xinyuan County, Xinjiang, China	43.248167°N, 84.011416°E	1882 m	4 June 2020	J.Qiu XY-002 (XJA)
G. granulosa (2)	Burqin County, Xinjiang, China	48.429694°N, 87.207108°E	1984 m	9 June 2021	J.Qiu & M.S.Lin L-023 (XJA)
G. granulosa (3)	Yuming County, Xinjiang, China	46.191895°N, 82.936688°E	709 m	28 April 2021	J.Qiu Yu-004 (XJA)
G. jaeschkei (1)	Tekes County, Xinjiang, China	43.411647°N, 81.040648°E	2538 m	9 June 2020	J.Qiu TKS-003 (XJA)
G. jaeschkei (2)	Qapqal County, Xinjiang, China	42.993913°N, 82.213955°E	2929 m	17 July 2021	J.Qiu & M.S.Lin L-30 (XJA)
G. jaeschkei (3)	Urumqi City, Xinjiang, China	43.119812°N, 86.855626°E	3492 m	6 June 2022	M.S.Lin & J.L.Li L-069 (XJA)
G. jaeschkei (4)	Bole City, Xinjiang, China	44.519168°N, 81.249635°E	2095 m	24 June 2022	J.L.Li L-070 (XJA)
G. jensii	Urumqi City, Xinjiang, China	43.783443°N, 87.544818°E	1002 m	8 April 2021	J.Qiu & M.S.Lin L-005 (XJA)
G. nigra (1)	Urumqi City, Xinjiang, China	43.783141°N, 87.544363°E	995 m	2 April 2021	J.Qiu & M.S.Lin L-003 (XJA)
G. nigra (2)	Urumqi City, Xinjiang, China	43.813774°N, 88.994927°E	1837 m	18 April 2021	J.Qiu & M.S.Lin L-012 (XJA)
G. nigra (3)	Urumqi City, Xinjiang, China	43.924234°N, 87.817345°E	916 m	5 April 2021	J.Qiu & M.S.Lin L-026 (XJA)
G. nigra (4)	Xinyuan County, Xinjiang, China	43.778801°N, 83.456579°E	1400 m	9 April 2021	X.J.Shi L-027 (XJA)
G. nigra (5)	Burqin County, Xinjiang, China	48.430593°N, 87.210654°E	1988 m	9 June 2021	J.Qiu & M.S.Lin L-024 (XJA)
G. nigra (6)	Yuming County, Xinjiang, China	45.842106°N, 82.525295°E	1676 m	28 April 2021	J.Qiu & M.S.Lin Yu-001 (XJA)
G. nigra (7)	Yuming County, Xinjiang, China	45.976943°N, 82.886943°E	1908 m	28 April 2021	J.Qiu & M.S.Lin Yu-008 (XJA)
G. nigra (8)	Urumqi City, Xinjiang, China	43.706494°N, 87.639277°E	1012 m	10 April 2022	J.Qiu & J.L.Li L-031 (XJA)
G. nigra (9)	Shihezi City, Xinjiang, China	44.188091°N, 86.088827°E	664 m	12 April 2022	J.Qiu & J.L.Li L-036 (XJA)
G. nigra (10)	Fukang City, Xinjiang,	43.897795°N,	2357 m	13 April 2022	J.Qiu & J.L.Li

	China	88.118855°E			L-037 (XJA)
G. neopopovii	Huocheng County, Xinjiang, China	44.480000°N, 81.170000°E	2100 m	19 May 2021	X.J. Ge Gexj-21019 (XJA)
G. kunawurensis (1)	Urumqi City, Xinjiang, China	43.785813°N, 87.545323°E	997 m	29 April 2021	J.Qiu & M.S.Lin L-015 (XJA)
G. kunawurensis (2)	Shihezi City, Xinjiang, China	44.194476°N, 86.079483°E	664 m	12 April 2022	J.Qiu & J.L.Li L-035 (XJA)
G. kunawurensis (3)	Shawan City, Xinjiang, China	44.235896°N, 85.821398°E	903 m	20 April 2022	J.Qiu & J.L.Li L-052 (XJA)
G. kunawurensis (4)	Yining City, Xinjiang, China	43.817749°N, 81.902141°E	880 m	22 April 2022	J.Qiu & J.L.Li L-057 (XJA)
G. stepposa (1)	Urumqi County, Xinjiang, China	43.516102°N, 87.447984°E	1559 m	10 April 2022	J.Qiu & J.L.Li L-032 (XJA)
G. stepposa (2)	Hutubi County, Xinjiang, China	43.821214°N, 86.429608°E	1160 m	20 April 2022	J.Qiu & J.L.Li L-040 (XJA)
G. tenera (1)	Yining City, Xinjiang, China	43.625463°N, 82.128763°E	600 m	29 April 2020	J.C.Chi Chijc1907 (XJA)
G. tenera (2)	Nilka County, Xinjiang, China	43.724538°N, 82.070252°E	1033 m	22 April 2022	J.Qiu & J.L.Li L-041 (XJA)

Appendix 2. A one-way ANOVA results of species with more than two populations.

Species	*Gagea alberti*	*G. altaica*	*G. bulbifera*	*G. divaricata*	*G. fedtschenkoana*	*G. filiformis*	*G. fragifera*
df	4	2	6	2	5	4	2
F	0.756	0.953	0.409	1.333	1.908	1.991	1.052
p	0.555	0.389	0.872	0.269	0.095	0.099	0.354

Appendix 2. (Continued).

Species	*Gagea granulosa*	*G. jaeschkei*	*G. nigra*	*G. kunawurensis*	*G. stepposa*	*G. tenera*
df	2	3	9	3	1	1
F	1.236	2.154	0.986	0.623	2.732	0.131
p	0.296	0.097	0.452	0.602	0.104	0.718
